# Supplementary material for: Updating estimates of Plasmodium knowlesi malaria risk in response to changing land use patterns across Southeast Asia
Source: medRxiv. 2023 Aug 8:2023.08.04.23293633. Preprint. [Version 1] doi: 10.1101/2023.08.04.23293633 (PMC10441477; doi:10.1101/2023.08.04.23293633)
Supplement: Supplement 1 [file NIHPP2023.08.04.23293633v1-supplement-1.pdf]

medRxiv preprint doi: https://doi.org/10.1101/2023.08.04.23293633; this version posted August 8, 2023. The copyright holder for this preprint (which was not certified by peer review) is the author/funder, who has granted medRxiv a license to display the preprint in perpetuity.
It is made available under a CC-BY 4.0 International license .

Supplementary Information

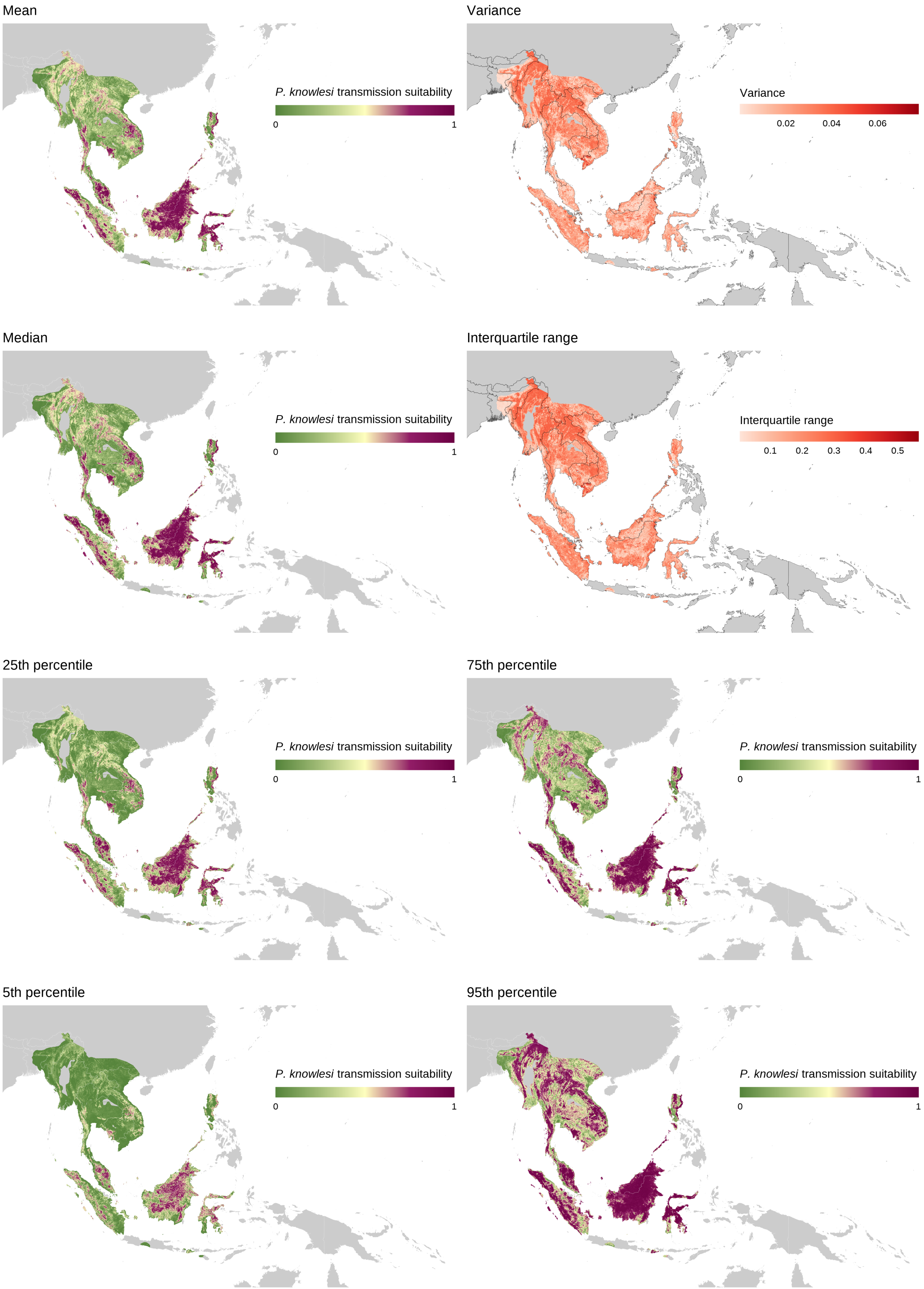

Figure S1: Summary statistics for modelled transmission suitability across Southeast Asia, calculated across the set of 500 bootstraps. Results are only displayed where an area is in the range of both a vector and reservoir species necessary for transmission (see Methods).

medRxiv preprint doi: https://doi.org/10.1101/2023.08.04.23293633; this version posted August 8, 2023. The copyright holder for this preprint (which was not certified by peer review) is the author/funder, who has granted medRxiv a license to display the preprint in perpetuity.
It is made available under a CC-BY 4.0 International license .

	Human	Macaque	Mosquito	Totals	
Country/Region	n	n	n	2020	(2015)
Brunei	0	0	0	0	(6)
Cambodia	5	0	0	5	(6)
Indonesia (Total)	44	1	0	45	(5)
Kalimantan Selatan	0	0	0	0	(3)
Kalimantan Tengah	1	0	0	1	(2)
Lampung	0	1	0	1	(0)
Nanggroe Aceh Darusalam	36	0	0	36	(0)
Sumatera Utara	7	0	0	7	(0)
Laos	3	0	0	3	(1)
Malaysia (Total)	181	8	12	201	(183)
Johor	3	0	0	3	(3)
Kedeh	2	0	0	2	(1)
Kelantan	5	0	0	5	(17)
Melaka	2	0	0	2	(1)
Negeri	2	0	2	4	(3)
Pahang	3	0	1	4	(11)
Perak	3	0	1	4	(1)
Pulau Pinang	1	0	0	1	(1)
Sabah	127	7	0	134	(60)
Sarawak	22	1	5	28	(52)
Selangor	7	0	3	10	(30)
Terengganu	1	0	0	1	(2)
W.P. Kuala Lumpur	3	0	0	3	(0)
W.P. Labuan	0	0	0	0	(1)
Myanmar	2	0	0	2	(3)
Philippines	1	0	2	3	(7)
Singapore	0	0	0	0	(6)
Thailand	4	0	0	4	(32)
Vietnam	1	0	0	1	(11)

Table S1: The number of human, macaque and mosquito samples in the occurrence database produced by the 2015 literature review, with samples in Indonesia and Malaysia shown stratified by region (province, state or territory). Total counts are shown for records from both the 2020 literature review and 2015 literature review.

medRxiv preprint doi: https://doi.org/10.1101/2023.08.04.23293633; this version posted August 8, 2023. The copyright holder for this preprint (which was not certified by peer review) is the author/funder, who has granted medRxiv a license to display the preprint in perpetuity.
It is made available under a CC-BY 4.0 International license .

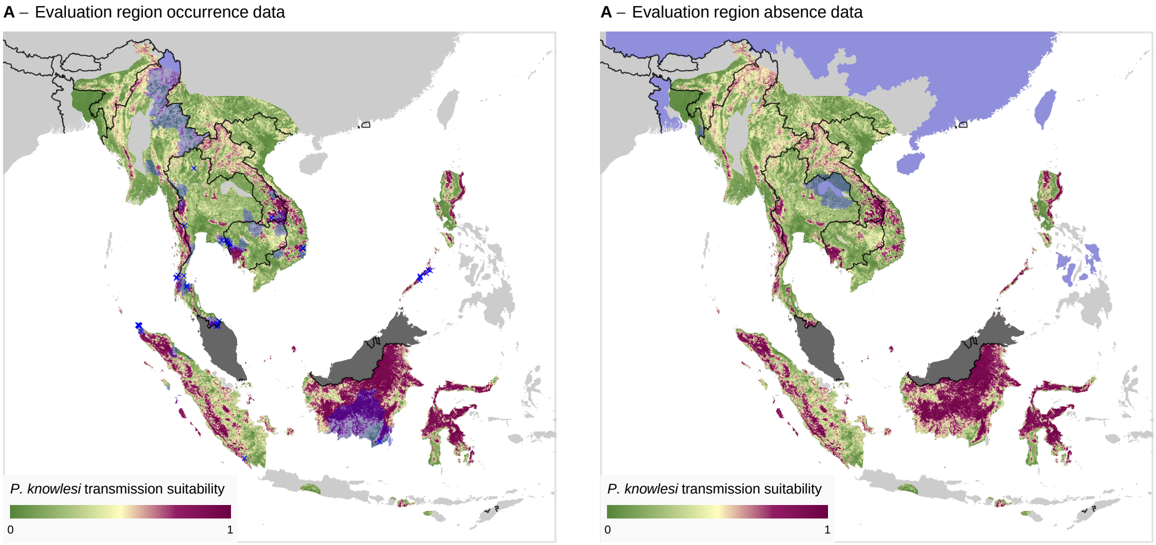

Figure S2: Predicted mean transmission suitability with overlay of infection occurrence data across the evaluation region. Results are only displayed where an area is in the range of both a vector and reservoir species necessary for transmission (see Methods). **A**: Predicted transmission suitability with infection occurrence polygons and points in blue. **B**: Predicted transmission suitability with infection absence polygons in blue.

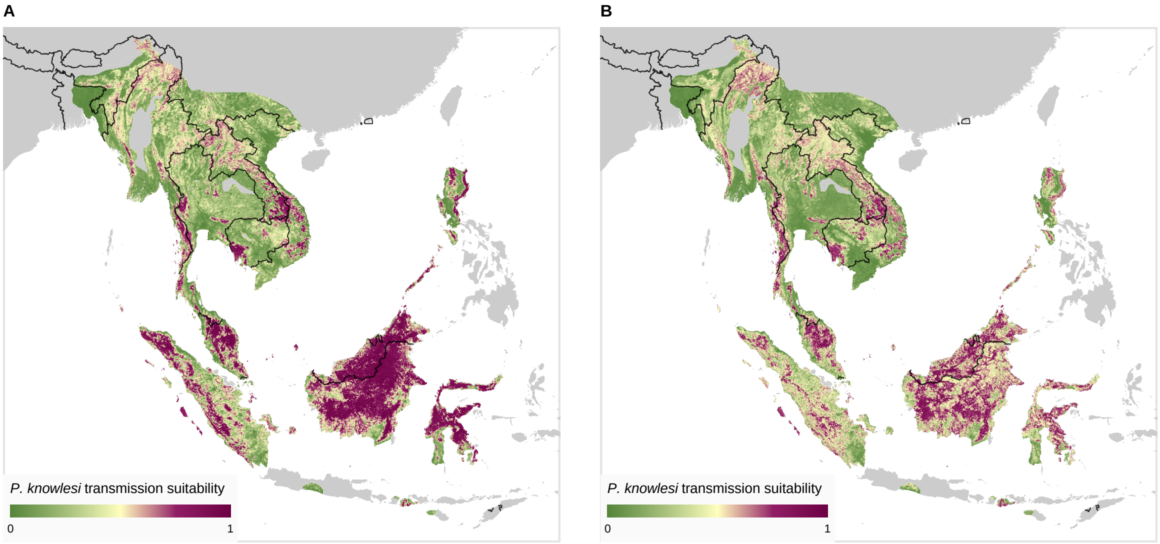

Figure S3: Comparison of the modelled mean transmission suitability value between the current work, with data up to 2020 (**A**), and the values presented in the 2015 model [31] (**B**). Note that the absolute value of predictions are not necessarily comparable given differences in model specification and training data. Results are only displayed where an area is in the range of both a vector and reservoir species necessary for transmission (see Methods).

medRxiv preprint doi: https://doi.org/10.1101/2023.08.04.23293633; this version posted August 8, 2023. The copyright holder for this preprint (which was not certified by peer review) is the author/funder, who has granted medRxiv a license to display the preprint in perpetuity.
It is made available under a CC-BY 4.0 International license .

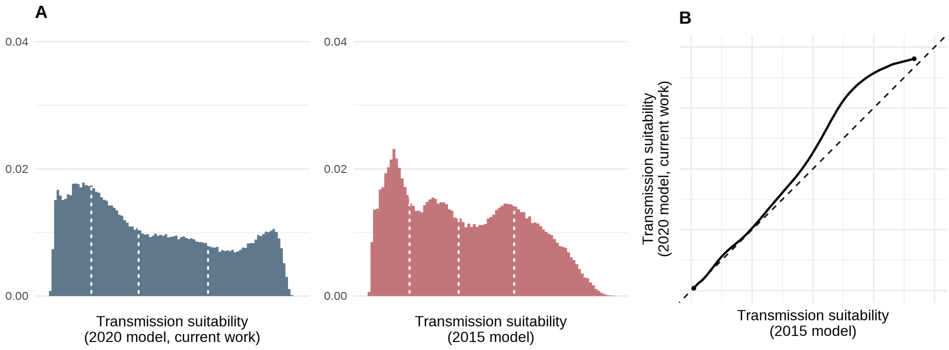

Figure S4: Histograms (**A**) and quantile-quantile plot (**B**) comparing the distributions of mean predicted transmission suitability for the 2015 and 2020 models of *P. knowlesi* transmission risk. Histograms are presented on a relative x-axis (ranging from minimal to maximal predicted mean risk), with quartiles of predicted risk displayed as dashed vertical lines.

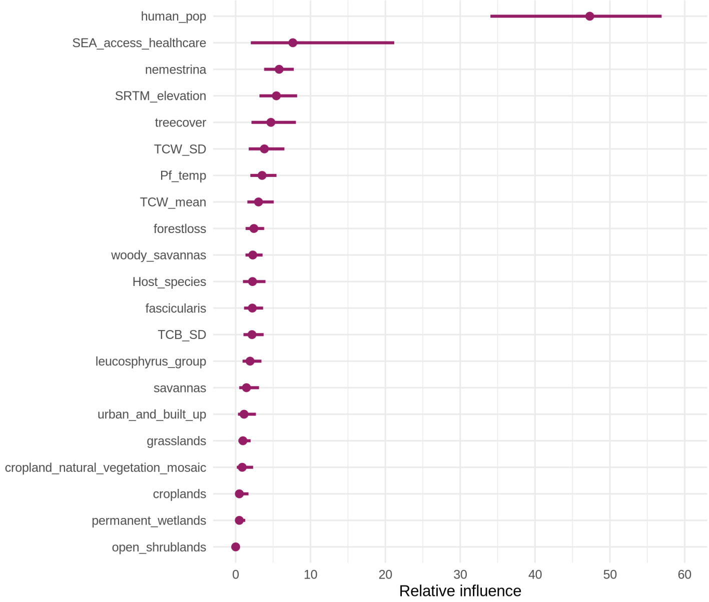

Figure S5: Relative influence scores for each covariate, calculated for each bootstrap, with points and lines representing median values and 95% confidence intervals respectively.

medRxiv preprint doi: https://doi.org/10.1101/2023.08.04.23293633; this version posted August 8, 2023. The copyright holder for this preprint (which was not certified by peer review) is the author/funder, who has granted medRxiv a license to display the preprint in perpetuity.
It is made available under a CC-BY 4.0 International license .

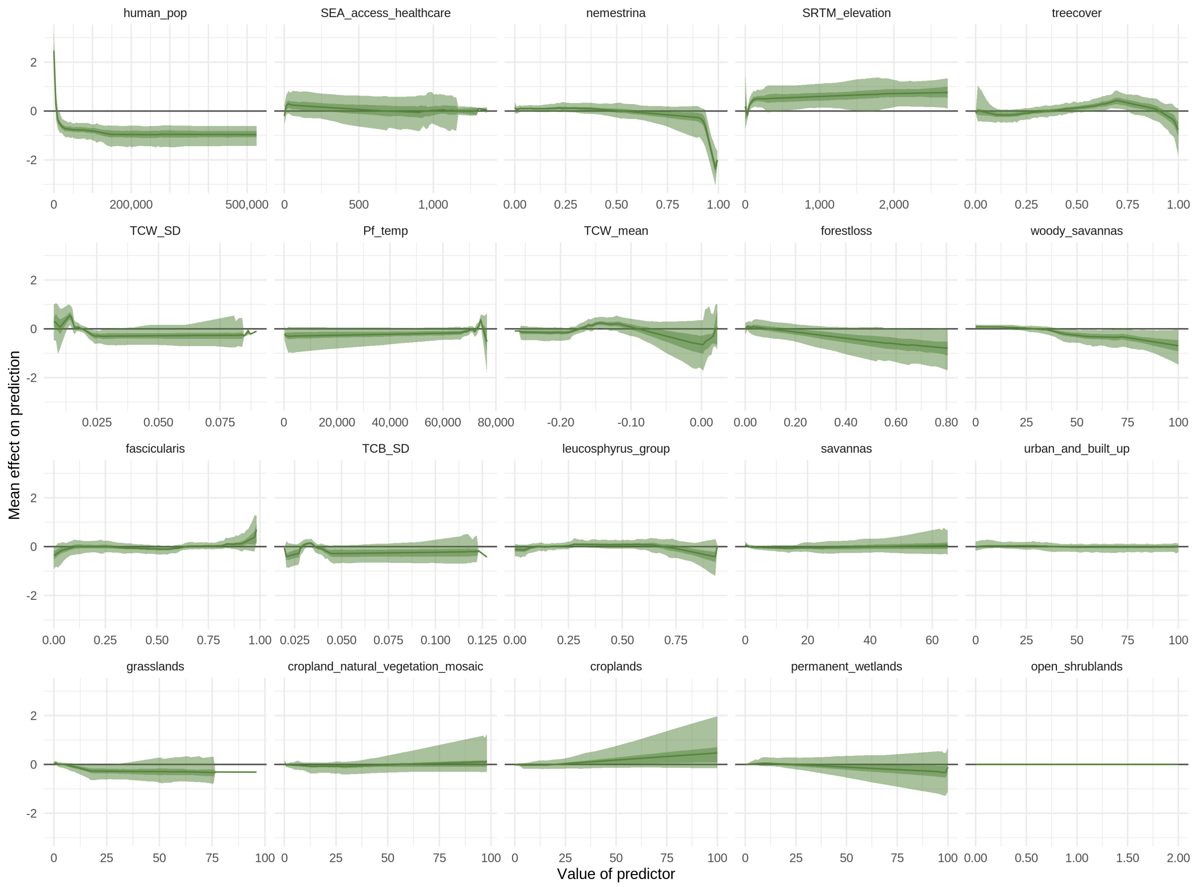

Figure S6: Accumulated local effect (ALE) plots for each covariate, indicating the mean effect of changing a covariate's value upon the prediction (on logistic scale) across the range of that covariate. The ALE values are calculated for each bootstrap, with the median value, 50% and 95% confidence intervals presented as lines, darker shaded regions and lighter shaded regions respectively.
